# A Manual of Dietetics

**Published:** 1889-03

**Authors:** 


### BOOK NOTICES AND REVIEWS.

A MANUAL OF DIETETICS. By W. B. Pritchard, M. D. Pages 87. Price, 50 cents. The Dietetic Publishing Company, New York city.

This little volume is a compendium of useful information upon the management of infants and also for the proper selection of diet for the sick. The style is simple, technical terms being avoided as much as possible; it is a volume for the laity rather than for the professional man.

